# Seroprevalence of *Toxoplasma gondii* infection among patients with hand, foot and mouth disease in Henan, China: a hospital-based study

**DOI:** 10.1186/s40249-015-0088-3

**Published:** 2015-12-10

**Authors:** Shuai Wang, Chunwei Lan, Luwen Zhang, Haizhu Zhang, Zhijun Yao, Dong Wang, Jingbo Ma, Jiarong Deng, Shiguo Liu

**Affiliations:** Department of Human Parasitology, Xinxiang Medical University, Xinxiang, Henan 453003 PR China; First People’s Hospital of Pingdingshan, Pingdingshan, Henan 467000 PR China

**Keywords:** Toxoplasma gondii, Hand, Foot and mouth disease, Seroprevalence, Enzyme-linked immunosorbent assay, China

## Abstract

**Background:**

The prevalence of infection with *Toxoplasma gondii* (*T. gondii*) in humans has been increasing in China due to the growing number of cats in the country. Hand, foot and mouth disease (HFMD) is a serious public health issue in China and still one of the leading causes of child mortality. However, little is known about the epidemiology of *T. gondii* infection among HFMD patients.

**Methods:**

A case–control study of 281 HFMD patients from the First People’s Hospital of Pingdingshan in Pingdingshan city, Henan province, central China, and 222 controls from Pingdingshan city was conducted. Anti-*T. gondii* antibodies were serologically detected using the enzyme-linked immunosorbent assay.

**Results:**

We found that the overall anti-*T. gondii* immunoglobulin G (IgG) antibody prevalence among HFMD patients was 12.46 %, which was significantly higher than that in clinically healthy children (1.80 %). The highest *T. gondii* seroprevalence was detected in critical cases (22.58 %), followed by severe cases (11.50 %), and the lowest was detected in mild cases (8.33 %).

**Conclusion:**

The present study is the first survey of *T. gondii* seroprevalence among HFMD patients in China; 12.46 % were defined as seropositive. It is imperative that improved integrated measures are taken to prevent and control *T. gondii* infection among HFMD patients.

**Electronic supplementary material:**

The online version of this article (doi:10.1186/s40249-015-0088-3) contains supplementary material, which is available to authorized users.

## Multilingual abstracts

Please see Additional file 1 for translations of the abstract into the six official working languages of the United Nations.

## Background

Hand, foot and mouth disease (HFMD) is a common viral illness mainly caused by coxsackievirus A16 (Cox A16) and enterovirus 71 (EV71) [1, 2] infections. Children under five years of age are more susceptible to HFMD, as over 50 % of children in this age bracket lack the neutralizing antibodies that protect against EV71 and Cox A16 infections [1, 3, 4].

Symptoms of HFMD include fever, headache and poor appetite, followed by an intensely sore throat and a rash with very small blisters on the hands, feet and diaper area. A small number of children suffer complications such as myocarditis, pulmonary edema and aseptic meningoencephalitis, which can be fatal [5–7]. Occasionally, adults can contract HFMD [1, 8].

Since the 1980s, a number of severe HFMD outbreaks have been documented and HFMD remains a significant public health challenge, especially in the Asia-Pacific region [9–13]. Since the initiation of the national surveillance for HFMD in China in 2008, the incidence of reported cases has sharply increased, with more than seven million cases reported, including approximately 2,500 deaths [11]. From 1 January 2008 to 31 December 2013, a total of 400,264 HFMD cases have been reported in the Henan province alone, including 22,309 severe and 141 fatal cases [11].

According to the guidelines of the National Health and Family Planning Commission (NHFPC) of the People’s Republic of China for diagnosis and treatment of HFMD (2010 edition, http://www.nhfpc.gov.cn/), HFMD cases are classified as mild, severe or critical. In mild cases, a skin rash appears on the hands, feet, mouth or buttocks of the patient, with or without fever. In severe cases, in addition to the above, patients can experience deflated moods or lethargy; become easily frightened or delirious; develop headaches; suffer from vomiting, limb shaking, myoclonus, nystagmus, ataxia, eye movement disorders, weakness or acute flaccid paralysis; and/or experience convulsions. There are also visible signs of meningeal irritation, and tendon reflexes are diminished or absent. In critical cases, at least one of the following has to occur: frequent convulsions, coma and brain herniation; dyspnea, cyanosis, bloody foam sputum, pulmonary rales, etc.; or shock and other signs of circulatory insufficiency.

*Toxoplasma gondii* (*T. gondii*) is a protozoan parasite that can infect virtually all warm-blooded animals, including humans. It has been estimated that one-third of the world’s population has been infected with this parasite [14, 15]. The prevalence of infection with *T. gondii* in humans has been increasing in China due to the growing number of cats in the country [16]. Although in immunocompetent individuals, most *T. gondii* infections are asymptomatic, the infection can lead to serious diseases among immunocompromised patients such as HIV-positive and cancer patients, and transplant recipients [17–19].

Hand, foot and mouth disease is a serious public health issue in China and still one of the leading causes of child mortality. Numerous studies have shown that Th1/Th2 and Th17/Treg imbalances exist in HFMD patients [20]. Children with HFMD caused by the EV71 infection can suffer from functional disorders of cell, humoral and innate immunity [21]. Any of these functional disorders can increase the chance of infection with opportunistic pathogens such as *T. gondii* [22]. Yet, epidemiological knowledge about the prevalence of *T. gondii* infection among HFMD patients is unavailable in China. The present study is the first to estimate the prevalence of *T. gondii* infection among HFMD patients in China.

## Methods

### Sample collection

A total of 281 clinically diagnosed HFMD patients (24 mild cases, 226 severe cases and 31 critical cases) from the First People’s Hospital of Pingdingshan in Pingdingshan city, Henan province, central China, were enrolled in the study from March to April 2014. The HFMD patients were classified into mild, severe or critical cases, according to the guidelines of the NHFPC of the People’s Republic of China for diagnosis and treatment of HFMD (2010 edition, http://www.nhfpc.gov.cn/). Demographic data such as gender and age were obtained. Two hundred and twenty-two control subjects, matched with HFMD patients by age, gender and residence, were also included in the study. They were clinically healthy children without EV71, CoxA16 or other enterovirus infections, all from Pingdingshan city.

Samples of approximately 4 ml of venous blood were taken from each patient and the control subjects with informed consent. All blood samples were labelled individually and cooled using ice packs to maintain the temperature at 4 °C during transportation to the laboratory.

### Serum sample preparation

Blood samples were centrifuged and sera were recovered and transferred to 1.5 ml Eppendorf Tubes™. The serum samples were stored at − 80 °C until tested for *T. gondii* antibodies.

### *Toxoplasma*-IgG assessment

Immunoglobulin G (IgG) antibodies of *T. gondii* were detected using a commercially manufactured enzyme-linked immunosorbent assay (ELISA) kit (Zhuhai S.E.Z. Haitai Biological Pharmaceuticals Co., Ltd, Zhuhai, China). The manufacturer’s instructions were followed to conduct the procedure. In brief, test serum (1:100 dilution) was added to each well in the coated plate and incubated for 30 minutes at 37 °C. After additional washing with a washing solution (PBS containing 0.05 % Tween20, PBST), 50 μL of peroxidase-conjugated anti-human IgG was added to the wells and incubated at 37 °C for 30 minutes. This was followed by three washes using the washing solution. The colour reactions were developed by adding 50 μL “A” solution (H_2_O_2_) and 50 μL “B” solution (3,3′,5,5′-Tetramethylbenzidine) at 37 °C for 10 minutes, and then the reaction was stopped by adding 50 μL of stopping solution (0.5 ml/L H_2_SO_4_). Microplates were read at an optical density (OD) of 450_nm_ in the Model 550 microplate ELISA reader (Bio-Rad, Hercules, CA, USA), and ratios (OD450 nm value of serum sample/OD450 nm value of negative control) were calculated after correcting for the OD450 nm value of the blank control (without serum and peroxidase-conjugated anti-human IgG). Test serum samples were considered positive when the ratio was ≥2.1.

### Statistical analysis

Statistical analysis was performed using the SPSS version 20 software for Windows (SPSS Inc., Chicago, Illinois, USA). The chi-square test was used to perform statistical analyses of *T. gondii* prevalence using different variables. Differences were considered statistically significant if *p* < 0.05.

### Ethical statement

The study was reviewed and approved by the Ethical Review Committee of the Xinxiang Medical University (reference no. 2013008). Written informed consent was obtained from all participants.

## Results

As shown in Table 1, anti-*T. gondii* IgG antibodies were detectable in the sera of 35 HFMD cases (35/281), with an overall seroprevalence of 12.46 %. For clinically healthy children, a total of four (1.80 %) of the 222 samples were positive for anti-*T. gondii* IgG antibodies. The seropositive rate of the former was significantly higher than that of the latter (*X*^2^ = 19.681, *p* <0.001).

**Table 1:** Seroprevalence of *Toxoplasma gondii* infection in the study populations in China

Variable	HFMD patients			Control subjects			HFMD patients *vs* control subjects	
	No. examined	No.of positive	Prevalence (%)	No. examined	No.of positive	Prevalence (%)	*X* ^2^	*P*-value
Gender								
Male	187	23	12.30	130	4	3.08	8.371	0.004
Female	94	12	12.77	92	0	0	12.555	<0.001
Age (year)								
<1	124	11	8.87	64	0	0	4.527	0.033
1 ~ 2	87	14	16.09	56	0	0	9.989	0.002
2 ~ 3	44	6	13.64	52	0	0	5.415	0.02
>3	26	4	15.38	50	4	8.00	0.362	0.548
Total	281	35	12.46	222	4	1.80	19.681	<0.001

In HFMD cases, the seroprevalence of *T. gondii* in males was 12.30 % (23/187) and 12.77 % (12/94) in females. No significant difference between genders was observed (*X*^2^ = 0.012, *p* = 0.911). The highest seroprevalence of *T. gondii* infection was found among children in the age group of one to two years (16.09 %, 14/87) and the lowest was found among children in the age group of < one year (8.87 %, 11/124). However, the difference between age groups was not significantly significant (*p* >0.05) (see Table 1).

Table 2 shows the clinical diagnosis data of HFMD patients based on the severity of their disease. The seroprevalence of *T. gondii* infection was highest in critical cases (22.58 %, 7/31), followed by severe cases (11.50 %, 26/226), and was the lowest in mild cases (8.33 %, 2/24). However, no statistically significant differences were observed among the three groups (*X*^2^ = 3.476, *p* = 0.176).

**Table 2:** Clinical diagnosis and seroprevalence of *T. gondii* in HFMD patients in China

Clinical classification	Patients with anti-*T. gondii* antibodies		
	No.examined	No.of positive	Prevalence (%)
Mild cases	24	2	8.33^a^
Severe cases	226	26	11.50^a^
Critical cases	31	7	22.58^a^
Total	281	35	12.46

Values bearing a different superscript letter (a) within a column differ significantly from one another (*p* < 0.05)

## Discussion

Infection with *T. gondii* in humans is common all around the world, with prevalence rates varying depending on the environment, people’s eating habits and age [23]. The two nation-wide surveys carried out in 1995 and 2004 reported that the prevalence of *T. gondii* infection in the Chinese population was around 7 % [24]. In 2010, the infection rate of *T. gondii* in the Chinese population increased to 12.3 % [25].

In the present study, 12.46 % (35/281) of HFMD patients were seropositive for *T. gondii*. This is higher than that reported in HIV-positive patients (3.5–7.7 %) [26, 27], similar to the general infection rate of *T. gondii* (12.3 %) [25], and lower than that observed in female sterility patients (15.9 %) [28], patients in intensive care units (18.78 %) [23], psychiatric patients (17.30 %) [29], cancer patients (35.56 %) and dialysis patients (27.3 %) [30, 31] in China.

Additionally, the seropositive rate of *T. gondii* infection among HFMD patients was significantly higher than that of clinically healthy children (1.80 %). Children with HFMD caused by the EV71 infection can suffer from functional disorders of cell, humoral and innate immunity [20, 21], which can reduce the body’s resistance to opportunistic pathogens such as *T. gondii* and predispose them to develop secondary toxoplasmosis. Latent *T. gondii* infections could also be activated. However, whether the prevalence of *T. gondii* infection can increase the risk of contracting HFMD and affect the disease outcome of HFMD patients requires further study.

In the present study, female HFMD patients had a higher prevalence than male patients, although the difference was not significant (*p* > 0.05). Gender was thus not significantly associated with the presence of anti-*T. gondii* antibodies (*P* >0.05), which was consistent with other reports [32, 33].

Rates of *T. gondii* infection increased with age in this study, however, the difference between age groups was not significantly significant (*p* >0.05). A hypothesis would be that the increase is a reflection of increasing ‘exposure years’ as the children get older [32]. The results provided further evidence for the increased risk of *T. gondii* infection with acquisition of age. Seroprevalence of *T. gondii* infection was highest in critical HFMD cases, followed by severe cases, and the lowest was detected in mild cases, with percentages of CD4^+^, CD8^+^ T and CD16^+^56^+^ cells (natural killer cells) continuously decreasing as the disease worsens [21]. Additionally, CD4^+^ and CD8^+^ T cells and natural killer cells play important roles in the process of anti-*T. gondii* infection [34, 35]. These findings might explain why the probability of seroprevalence with *T. gondii* infection increases with the severity of the HFMD disease. With respect to the increased numbers of HFMD patients worldwide, toxoplasmosis should be considered as potentially associated with HFMD in humans.

## Conclusion

The present study revealed for the first time the seroprevalence of *T. gondii* infection among HFMD patients in the Henan province, China. Further studies should be conducted to estimate the prevalence of *T. gondii* infection among HFMD patients in other provinces of China, with increased sample sizes.

## Additional files

Additional file 1:
**Multilingual abstracts in the six official working languages of the United Nations.** (PDF 341 kb)

## Abbreviations

Cox A16: coxsackievirus A16
ELISA: enzyme-linked immunosorbent assay
EV71: enterovirus 71
HFMD: hand, foot and mouth disease
IgG: immunoglobulin G
NHFPC: National Health and Family Planning Commission
OD: optical density
T. gondii: Toxoplasma gondii
