# Supplementary material for: Seroprevalence of Toxoplasma gondii infection among patients with hand, foot and mouth disease in Henan, China: a hospital-based study
Source: Infect Dis Poverty. 2015 Dec 10;4:53. doi: 10.1186/s40249-015-0088-3 (PMC4675030; doi:10.1186/s40249-015-0088-3)
Supplement: Additional file 1: — Multilingual abstracts in the six official working languages of the United Nations. (PDF 341 kb) [file 40249_2015_88_MOESM1_ESM.pdf]

Translation of the abstract into the six official working languages of the United Nations

الانتشار المصلي لعدوى التوكسوبلازما التوكسوبلازما بين المرضى المصابين بداء اليد والقدم والفم في خنان، الصين: دراسة في المستشفيات

شواي وانغ، تشونوي لان، جياورنغ دنغ، لوين تشانغ، هيزو تشانغ، تشي جون ياو، دونغ وانغ، جينغ بو ما، شينغو ليو

ملخص

معلومات عامة: يتزايد انتشار عدوى **التوكسوبلازما التوكسوبلازما** ***(التوكسوبلازما)*** بين البشر في الصين نتيجة العدد المتنامي من القطط في البلاد. يمثل داء اليد والقدم والفم (HFMD) مرضاً خطيراً يهدد الصحة العامة في الصين ومازال واحداً من الأسباب الرئيسية لوفيات الأطفال. ورغم ذلك فليس هناك الكثير مما يُعرف عن انتشار عدوى **التوكسوبلازما التوكسوبلازما** بين المصابين بداء اليد والقدم والفم.

الاكتشافات: أُجريت دراسة الحالات والشواهد على 281 مريضاً مصاباً بداء اليد والقدم والفم في مستشفى فيرست بيبول في بينغدينغشان بمدينة بينغدينغشان في مقاطعة خنان بوسط الصين و222 من الشواهد من مدينة بينغدينغشان. تم الكشف مصلياً عن الأجسام المضادة **التوكسوبلازما التوكسوبلازما** باستخدام الفحص المناعي المرتبط بالإنزيم. وجدنا أن معدل الانتشار العام لأجسام الغلوبولين المناعي (IgG) المضادة **التوكسوبلازما التوكسوبلازما** بين المصابين بداء اليد والقدم والفم يبلغ 12.46%، وهو ما كان أعلى بكثير من المعدل في الأطفال الأصحاء سريرياً (1.80٪). تم اكتشاف أعلى معدل انتشار مصلي **التوكسوبلازما التوكسوبلازما** في حالات حرجة (22.58%)، يليه حالات وخيمة (11.50%)، وأقل معدل في حالات خفيفة (8.33%).

الاستنتاج: الدراسة الحالية هي أول استقصاء عن الانتشار المصلي **التوكسوبلازما التوكسوبلازما** بين المصابين بداء اليد والقدم والفم في الصين، وتم تحديد 12.46% من الحالات بإيجابية المصل. لا بد من اتخاذ إجراءات متكاملة ومحسنة لمنع عدوى **التوكسوبلازما التوكسوبلازما** والسيطرة عليها بين المصابين بداء اليد والقدم والفم.

Translated from English version into Arabic by Ran Abdel Rahman, through

TRANSLATORS
WITHOUT BORDERS

河南省手足口病患者弓形虫感染情况调查

王帅，兰春伟，张露文，张海珠，姚志军，王东，马静波，邓家荣，刘世国*

摘要

目的：随着宠物猫饲养量的不断增加，中国人群的弓形虫感染率不断上升。在中国，手足口病仍然是一个严重的公共卫生问题，同时也是导致儿童死亡的主要病因之一。然而到目前为止，有关手足口病患者感染弓形虫的流行情况尚不清楚，为此进行了本研究。

方法：用酶联免疫吸附试验（ELISA）检测 281 例临床确诊的手足口病患者以及 222 名健康儿童血清中是否含有抗弓形虫 IgG 抗体。

结果：手足口病患者的弓形虫感染率为 12.46%（35/281），健康儿童的感染率 1.80%（4/281），前者的感染率显著高于后者(χ^2=19.681，p<0.01)；普通手足口病患者、重型手足口病患者和危重型手足口病患者的弓形虫感染率分别为 8.33%（2/24）、11.50%（26/226）和 22.58%（7/31），三组之间差别不具有统计学差异（χ^2 = 3.476，p＞0.05）。

结论：本研究首次调查了河南省手足口病患者的弓形虫感染情况，其中 12.46%的患者为弓形虫阳性，应引

起医务人员的足够重视。

Translated from English version into Chinese by Wang Shuai.

Séroprévalence de l'infection par *Toxoplasma gondii* parmi des patients atteints de la maladie pieds-mains-bouche au Henan, Chine : une étude en milieu hospitalier

Shuai Wang, Chunwei Lan, Jiarong Deng, Luwen Zhang, Haizhu Zhang, Zhijun Yao, Dong Wang, Jingbo Ma, Shiguo Liu

Résumé

Contexte : La prévalence de l'infection par *Toxoplasma gondii* (*T. gondii*) chez les êtres humains a augmenté en Chine en raison du nombre croissant de chats dans le pays. La maladie pieds-mains-bouche est un sérieux problème de santé publique en Chine et reste l'une des principales causes de mortalité infantile. On connait néanmoins peu de choses à propos de l'épidémiologie de l'infection par *T. gondii* parmi les patients atteints de cette maladie.

Observations : Nous avons mené une étude cas-témoin réunissant 281 patients atteints de la maladie pieds-mains-bouche provenant de l'Hôpital populaire de Pingdingchan, province de Henan, Chine centrale, et 222 contrôles de la ville de Pingdingchan. Des anticorps dirigés contre le *T. gondii* ont été détectés sérologiquement par l'utilisation de l'essai d'immuno-absorption enzymatique. Nous avons découvert que la prévalence globale de l'anticorps IgG dirigé contre le *T. gondii* parmi des patients atteints de la maladie pieds-mains-bouche s'élevait à 12,46 %, une proportion nettement plus élevée que chez des enfants sains d'un point de vue clinique (1,80 %). La séroprévalence du *T. gondii* la plus élevée a été détectée dans les cas critiques (22,58 %), suivie des cas sévères (11,50 %) et la séroprévalence la plus faible a été mesurée dans les cas légers (8,33 %).

Conclusion : La présente étude constitue la première enquête relative à la séroprévalence du *T. gondii* parmi des patients atteints de la maladie pieds-mains-bouche en Chine ; 12,46 % des cas étaient définis comme séropositifs. La prise de mesures intégrées et améliorées est impérative afin de prévenir et de contrôler l'infection par *T. gondii* parmi les patients atteints de la maladie pieds-mains-bouche.

Translated from English version into French by Eric Ragu, through

TRANSLATORS
WITHOUT BORDERS

Доминирование серотипа инфекции токсоплазма гонди (*Toxoplasma gondii)* у пациентов с заболеванием кистей рук, стоп и полости рта (HFMD) в провинции Хэнань, Китай: клиническое исследование

Шуай Ванг, Чунвей Лан, Джиаронг Денг, Лювень Жанг, Хайжу Жанг, Жиюнь Яо, Донг Ванг, Джинбо Ма, Шигуо Лью

Реферат

История вопроса: В Китае продолжает расти распространённость инфицирования людей *Toxoplasma gondii* (*T. gondii*) вследствие увеличения в стране количества котов. Заболевание кистей рук, стоп и полости рта (HFMD) является серьезной проблемой здравоохранения в Китае и одной из основных причин детской смертности. Однако мало известно об эпидемиологии инфекции *T. gondii* у больных HFMD.

Данные исследования: Было проведено исследование методом случай-контроль 281 больных с HFMD из Первого народного госпиталя в городе Пиндингшан провинции Хэнань в Центральном Китае и 222 лиц из контрольной группы из города Пиндингшан. Обнаружение антител*T. gondii* проводилось серологически методом твердофазного иммуноферментного анализа. Было установлено, что общий уровень распространения антител *T. gondii* - иммуноглобулина G (IgG) - у больных HFMD составлял 12,46%, что значительно превышает их уровень распространения у клинически здоровых детей (1,80%). Самое высокое доминирование серотипа *T. gondii* было выявлено в критических случаях заболевания (22,58%), затем в серьезных случаях (11,50%), а самое низкое в легких случаях заболевания (8,33%).

Выводы: Настоящее исследование является первым исследованием доминирования серотипа *T. gondii* среди больных HFMD в Китае; 12,46% были определены как серопозитивные. Крайне необходимо принять компклесные меры для предотвращения и контроля инфекции *T. gondii* у больных HFMD.

Translated from English version into Russian by Alena Hrybouskaya, through

TRANSLATORS
WITHOUT BORDERS

Seroprevalencia de la infección por *Toxoplasma gondii* en pacientes con fiebre aftosa en Henan, China: un estudio en hospital

Shuai Wang, Chunwei Lan, Jiarong Deng, Luwen Zhang, Haizhu Zhang, Zhijun Yao, Dong Wang, Jingbo Ma, Shiguo Liu

Resumen

Antecedentes: La prevalencia de la infección por *Toxoplasma gondii* (*T. Gondii)*) en seres humanos ha aumentado en China debido al aumento de la cantidad de gatos en el país. La fiebre aftosa es un serio problema de salud pública en China y continúa siendo una de las primeras causas de mortalidad infantil. Sin embargo, poco se conoce sobre la epidemiología de la infección por *T. gondii* en los pacientes con fiebre aftosa.

Hallazgos: Se llevó a cabo un estudio de casos y controles de 281 pacientes con fiebre aftosa del First People's Hospital of Pingdingshan en la ciudad de Pingdingshan, provincia de Henan, en el centro de China, y 222 controles de la ciudad de Pingdingshan. Los anticuerpos anti *T. gondii* se detectaron serológicamente mediante el uso de ensayo por inmunoabsorción ligado a enzimas. Encontramos en general que la prevalencia de anticuerpos

inmunoglobulina G (IgG) contra T. gondii en pacientes con fiebre aftosa fue de 12,46%, lo que es significativamente más elevada que en niños clínicamente sanos (1,80%). La seroprevalencia más alta de T. gondii se detectó en casos críticos (22,58%), seguido de casos severos (11,50%), y la menor se detectó en casos leves (8,33%).

Conclusión: El presente estudio es una encuesta inicial de la seroprevalencia de *T. gondii* en pacientes con fiebre aftosa en China. 12,46% fueron definidos como seropositivos. Es imperativo que se tomen mejores medidas integradas para prevenir y controlar la infección por *T. gondii* en pacientes con fiebre aftosa.

Translated from English version into Spanish by Maria Alejandra Aguada, through

TRANSLATORS
WITHOUT BORDERS
